# Hormonal requirements for effective induction of microspore embryogenesis in triticale (× *Triticosecale* Wittm.) anther cultures

**DOI:** 10.1007/s00299-014-1686-4

**Published:** 2014-09-27

**Authors:** Iwona Żur, Ewa Dubas, Monika Krzewska, Piotr Waligórski, Michał Dziurka, Franciszek Janowiak

**Affiliations:** The Franciszek Górski Institute of Plant Physiology, Polish Academy of Sciences, Niezapominajek 21, 30-239 Kraków, Poland

**Keywords:** Abscisic acid, Auxins, Cytokinins, Hormonal homeostasis, Microspore embryogenesis, Triticale

## Abstract

*****Key message***:**

**Effective microspore embryogenesis in triticale is determined by a specific hormonal homeostasis: low value of IAA/cytokinins, IAA/ABA and cytokinins/ABA ratios as well as proper endogenous/exogenous auxin balance, which favours androgenic structure formation and green plant regeneration ability.**

**Abstract:**

The concentration of plant growth regulators (PGRs): auxins (Auxs), cytokinins (CKs) and abscisic acid (ABA) was measured in anthers of eight DH lines of triticale (× *Triticosecale* Wittm.), and associated with microspore embryogenesis (ME) responsiveness. The analysis was conducted on anthers excised from control tillers at the phase optimal for ME induction and then after ME-initiating tillers treatment (21 days at 4 °C). In control, IAA predominated among Auxs (11–39 nmol g^−1^ DW), with IBA constituting only 1 % of total Auxs content. The prevailing isoforms of CKs were *cis* isomers of zeatin (121–424 pmol g^−1^ DW) and zeatin ryboside (*c*ZR, 146–432 pmol g^−1^ DW). Surprisingly, a relatively high level (10–64 pmol g^−1^ DW) of kinetin (KIN) was detected. Cold treatment significantly changed the levels of all analysed PGRs. The anthers of ‘responsive’ DH lines contained higher concentrations of IBA, *cis* and *trans* zeatin, *c*ZR and ABA, and lower amount of IAA and KIN in comparison with ‘recalcitrant’ genotypes. However, the effects of exogenous ABA, p-chlorophenoxyisobutyric acid (PCIB) and 2,3,5-triiodobenzoic acid treatments suggest that none of the studied PGRs acts alone in the acquisition of embryogenic competency, which seems to be an effect of concerted PGRs crosstalk. The initiation of ME required a certain threshold level of ABA. A crucial prerequisite for high ME effectiveness was a specific PGRs homeostasis: lower Auxs level in comparison with CKs and ABA, and lower CKs/ABA ratio. A proper balance between endogenous Auxs in anthers and exogenous Auxs supplied by culture media was also essential.

**Electronic supplementary material:**

The online version of this article (doi:10.1007/s00299-014-1686-4) contains supplementary material, which is available to authorized users.

## Introduction

Under specific circumstances usually occurring in in vitro cultures, plant microspores can enter an alternative developmental pathway known as microspore embryogenesis (ME) or androgenesis. As a result of this asexual form of development, microspores develop into haploid plants which, after genome diploidization, form totally homozygous doubled haploids (DHs), highly valued in many research areas and breeding practice (Kasha and Maluszynski 2003). From a practical point of view, due to potentially high effectiveness and relatively simple in vitro procedures, ME is the most promising and widely deployed method for DHs production. Many aspects of the process have been examined, but the precise mechanism of its initiation and factors determining high effectiveness have not been identified yet (Wędzony et al. 2009).

It is known that microspore reprogramming is triggered by a stress treatment, but the effectiveness of the process is controlled by an interaction of genetic and physiological factors. The most important is the genotype of the maternal plant though the great variation in ME efficiency that can be observed not only among various cultivars and lines but also among various inflorescences or even flowers suggests that the plant/tissue/cell physiological status can be a critical factor. One of the components of this physiological status is the homeostasis of plant growth regulators (PGRs), especially auxins (Auxs) and cytokinins (CKs), known as key signalling molecules that control plant growth and development. In in vitro, the concerted action of Auxs and CKs controls cell division and morphogenesis (Wędzony et al. 2009). These two phytohormone groups usually act antagonistically, but their effect is often modulated by the plant genome and tissue specificity (Moubayidin et al. 2009). Moreover, an altered homeostasis of PGRs is one of the most dynamic changes in response to stress conditions, initiating various signal transduction pathways (Kohli et al. 2013). The role of Auxs and CKs in ME has been examined and described by many authors (review in Wędzony et al. 2009), but the majority of studies focused on the optimization of exogenous PGRs content in the media used for in vitro culture. In a few cases when the endogenous PGRs level was analysed (Gorbunova et al. 2001; Lulsdorf et al. 2012), the obtained data suggested that ME effectiveness resulted from complex PGRs crosstalk, its interactions with the plant genotype and the environment, and equilibria established between endogenous and exogenous plant hormones. Besides Auxs and CKs, also abscisic acid (ABA), known as the main plant stress hormone, has been claimed to play a role in androgenesis-inducing signal transduction system (Maraschin et al. 2005; Żur et al. 2012). Some reports suggested the involvement of ABA in androgenesis induction and described its positive influence on the effectiveness of DHs production (Imamura and Harada 1980; Reynolds and Crawford 1996; Van Bergen et al. 1999; Wang et al. 1999; Guzman and Arias 2000).

The complexity of PGRs network, which could exist at the level of biosynthesis, distribution, gene expression or signalling pathways, renders the creation of an integrated model of ME-control crosstalk impossible at present. Precise analysis of changes in hormonal balance associated with ME initiation could produce new data and increase our knowledge about physiological background which determines high effectiveness of this process.

To gain a better understanding of the mechanisms controlling triticale ME, the endogenous level of PGRs: Auxs, CKs and ABA, was analysed in anthers at the phase optimal for ME initiation and then after low temperature treatment (3 weeks at 4 °C), which induced triticale microspore reprogramming. Eight DH lines of winter triticale (× *Triticosecale* Wittm.) with different androgenic responsiveness were selected as the objects of the study from the mapping population of 90 DH lines ‘Saka3006’×‘Modus’. To check how a disturbed Auxs/CKs/ABA ratio influences ME, cold treatment of tillers was combined with chemical treatments which should alter endogenous PGRs homeostasis in anthers. For this purpose, the influence of several substances, namely exogenous ABA, ABA biosynthesis inhibitor (NOR), anti-auxin (PCIB) and inhibitor of auxin transport (TIBA) on endogenous PGRs level in anthers and then on ME effectiveness in anther cultures was investigated. The same substances were also used as the supplement to the C17 medium to check how an altered PGRs balance during the induction phase influences ME effectiveness.

## Materials and methods

### Plant material

Eight winter triticale DH lines—four highly ‘responsive’ (DH44, DH28, DH101, DH47) and four ‘recalcitrant’ ones (DH19, DH72, DH119, DH144)—derived from the F1 generation of a cross between German inbred line ‘Saka 3006’ and Polish cv. ‘Modus’ were used in the study. Germinating triticale kernels were placed in perlite pre-soaked with Hoagland’s salt solution and vernalized for 7 weeks at 4 °C and 8/16 h (day/night) photoperiod. Vernalized seedlings were planted into pots containing a mixture of soil, de-acidified substrate peat and sand (2/2/1; v/v/v) and grown in a greenhouse at 25 °C with 16/8 h photoperiod.

### Protocol for anther culture

Tillers from donor plants were collected when the majority of microspores were at mid- to late-uninucleate stage of development. The tillers were placed in Hoagland’s salt solution and stored at 4 °C in the dark for 21 days. Then, under sterile conditions, cold-treated (CT) spikes were sterilized with 96 % ethanol, and the anthers were excised and transferred to modified C17 medium (Wang and Chen 1983 modified by Wędzony 2003) containing 1 mg l^−3^ Dicamba, 1 mg l^−3^ Picloram, 0.5 mg l^−3^ kinetin (KIN), 90 g l^−3^ maltose and 0.6 % agar (A1296 Sigma-Aldrich), pH 5.8. The cultures were incubated at 26 °C in the dark. Anthers excised from freshly cut (FC) tillers were used as the control. Starting from the 6th week of culture, embryo-like structures (ELS) bigger than 1 mm were transferred onto regeneration medium 190-2 (Zhuang and Xu 1983) supplemented with 30g l^−3^ sucrose, 0.5 mg l^−3^ KIN, 0.5 mg l^−3^ NAA and 0.6 % agar, pH 6.0. The cultures were kept at 26 °C with 16/8 h (day/night) photoperiod at 80–100 µmol m^−2^ s^−1^ light intensity. The passage was repeated three times at two-week intervals. The effectiveness of ME was expressed by several parameters: ELS/100A—the number of ELS produced per 100 anthers; R/100ELS—the number of regenerated plants per 100 ELS; GR/100ELS—the number of green regenerated plants per 100 ELS; R/100A—the total number of regenerated plants per 100 anthers; GR/100A—the number of green regenerated plants per 100 anthers. The parameters were calculated as the mean from ten biological replications where each 60 × 15 mm Petri dish containing about 100 anthers excised from one spike was considered as one replication.

### Samples preparation for Aux and CK HPLC analysis

Immediately after collection, the anthers were frozen in liquid nitrogen, lyophilized and homogenized while still frozen. Then, 50 mg of pulverized plant material was used for each sample. Auxs were extracted with a mixture of methanol/water/formic acid (15/4/1; v/v/v) according to Dobrev and Kamınek (2002) with modifications by Stefancic et al. (2007). Internal isotopic standard mixture consisting of deuterated IAA and KIN labelled with nitrogen ^15^N was added to each sample. This extract was fractionated with SPE columns Oasis MCX (Waters). Peak area of each compound was divided by peak area of appropriate internal standard, and thus transformed data were used for calibration table and for quantitation, thereby the efficiency of the process was automatically corrected.

### Quantification of Aux

Auxs fraction was eluted from SPE column with methanol, evaporated to dryness and reconstituted in 50 μl methanol. Samples prepared in this manner were analysed on HPLC column Supelco Ascentis RP-Amide (7.5 cm × 4.6 mm, 2.7 μm). Mobile phases were 0.1 % formic acid solution in water (solvent A) and acetonitrile/methanol (1/1) mixture. Gradient elution was applied under the flow rate of 0.5 ml/min. HPLC apparatus was Agilent Technologies 1260 equipped with Agilent Technologies 6410 Triple Quad LC/MS with ESI (Electrospray Interface). Two most abundant secondary ions were monitored (MRM—multiple reaction monitoring mode). One of them was used for quantification, whereas the second was used for additional confirmation of identity. The monitored ions were: indole-3-acetic acid (IAA)—m/z 176.1 primary, 130.3, 77.2 secondary; indolebutyric acid (IBA)—m/z 204.1 primary, 186.4, 130.3 secondary; D-IAA (deuterated IAA used as internal standard)—m/z 181.1 primary, 134.7, 81.4 secondary.

### Quantification of CK

CKs fractions were flushed out from SPE column after collecting Auxs. After washing with 0.35 M ammonia in water, CKs were eluted with 0.35 M ammonia in 60 % methanol. The collected fraction was evaporated to dryness, reconstituted in methanol and analysed using the same chromatographic system and columns as described above. The solvents, (a) water with 0.001 % acetic acid, and (b) acetonitrile with 0.001 % acetic acid were used at the flow rate of 0.5 ml min^−1^. The most abundant secondary ion was used for quantification of CKs, the other two were used for confirmation of their identity. The monitored ions were: zeatin (Z)—m/z 220.2 primary, 136.3, 202.3, 119.2 secondary; zeatin riboside (ZR)—m/z 352.4 primary, 220.3, 136.3, 119.2 secondary; KIN—m/z 216.2 primary, 81.3, 188.3, 148.3 secondary; KIN-^15^N (heavy nitrogen labelled KIN used as internal standard)—m/z 220.2 primary, 81.2, 192.3, 152.3 secondary.

### Quantification of ABA

Anthers with total weight of 0.3 g were isolated from FC and CT (21 days at 4 °C) tillers. Plant material was freeze dried and ground with ball mill MM400 (Retch, Germany) in 1.5 ml of cold distilled water. Then, the samples were placed in boiling water for 3 min and shaken overnight at 4 °C. The extracts were centrifuged for 20 min in a refrigerated centrifuge at 18,000×*g* (MPW-350R, Poland). ABA was measured in the supernatant using indirect enzyme-linked immunosorbent assay (ELISA) according to Walker-Simmons and Abrams (1991) with MAC 252 antibody (Babraham Technix, Cambridge, UK). Absorbance was measured by microplate reader Model 680 (Bio-Rad Laboratories, Inc.) at the wavelength of 405 nm. For each treatment, at least four independent ELISA measurements were made on two independent samples collected from four to six different plants.

### Immunocytochemical detection of IAA

#### Sampling

Samples of anthers were collected from FC and CT tillers of two DH lines (‘responsive’ DH28 and ‘recalcitrant’ DH19) of triticale.

#### IAA immunolocalization

The protocol for ‘whole-mount’ immunolocalization was adapted from Dubas et al. (2012) with substantial modification which enabled proper preservation of IAA by fixation and subsequent proper penetration of antibodies. To conserve the antigenicity of IAA, 1-ethyl-3-(dimethyl-aminopropyl)-carbodiimide (EDAC 3 %) in PBS (phosphate-buffered saline) was applied as the first pre-fixative (60 min). As the primary antibodies, anti-IAA-monoclonal antibodies were applied in 3 % BSA/MTSB (microtubule stabilizing buffer) at 4 °C overnight (anti-IAA antibody, A0855 clone 1E11-C11, purified immunoglobulin, dilution 1:1,000, Sigma). The secondary antibodies, anti-mouse IgG conjugated with alkaline phosphatase (Sigma) were added to each sample and incubated in blocking buffer for 3 h at 37 °C in darkness. Afterwards, the material was washed (six times for 10 min) with MTSB/0.025 % Triton, 50 mM 1,4-piperazinediethane sulfonic acid (PIPES, Sigma), 5 mM EGTA (Sigma), 5 mM MgSO_4_, pH 7.0, adjusted with 5 M KOH containing NaN_3_ (sodium azide 0.02 %) and with sterile Milli-Q (MQ) water. Washes were followed by 15-min staining with ready-to-use Western Blue (Promega) in the dark for 15–30 min. When blue/purple colour was observed, samples were rinsed and stored in PBS containing 0.02 % NaN_3_. Control experiments were performed by omitting the first antibody. For DNA counterstaining, the samples were incubated in PI (Propidium iodide, 0.1 % in H_2_O) for 15 min, washed in PBS, and embedded on slides in Citifluor glycerol (Citifluor Ltd. in glycerol, AF2, Enfield Cloisters).

#### Microscopic observation

Light and fluorescence microscopy was conducted with Nikon Eclipse E 600-microscope equipped with DIC (differential interference contrast) and digital camera DS-Ri1 (Nikon). Fluorescence was examined under filter EX 510–560/DM 580 BA/590 EF (PI). Images were acquired and processed using software programs including NIS-Elements (BR, AR 2.10 Laboratory Imaging System Ltd.) and CorelDRAW Essentials 10.0.

#### Modification of endogenous hormonal balance during androgenesis induction treatment (1) or in vitro induction phase (2)

Several substances, namely ABA—racemic mixture of (±) isomers (Sigma-Aldrich A1049); norflurazon (NOR)—ABA biosynthesis inhibitor (Supelco PS-1044); 2,3,5-triiodobenzoic acid (TIBA)—inhibitor of auxin transport (Sigma-Aldrich T5910); and p-chlorophenoxyisobutyric acid (PCIB)—anti-auxin blocking auxin receptors (Sigma Chemical Co. 197777) were supplied to Hoagland’s salt solution during cold tillers treatment for 3 or 21 days before anthers isolation. The substances were tested in concentrations: 10 µM ABA, 5 µM NOR, 10 µM TIBA, 5 µM PCIB, which had been selected on the basis of preliminary experiments and literature data. Dimethyl sulfoxide (DMSO), PCIB solvent in 0.1 % concentration was used as additional control.

In the second part of the experiment, 5 µM PCIB or 10 µM TIBA was added to modified C17 induction medium for the whole period of the induction phase. C17 medium deprived of synthetic Aux analogues (Dicamba, Picloram), containing only 0.5 mg l^−3^ KIN (noAuxs), was used as additional control.

‘Recalcitrant’ DH19 and ‘responsive’ DH28 were used in the study. Each spike was uniformly divided onto four probes (one row of anthers = 1 probe)—three were immediately frozen in liquid nitrogen and preserved at −60 °C up to the moment of HPLC analysis. One quarter of anthers was used for an estimation of DH line androgenic potential with the use of the anther culture method described above.

### Statistical analysis

The evaluation of data started with descriptive statistical analysis (mean, standard error). The normal distribution of scores was verified with Shapiro–Wilk test to validate the use of parametric tests. Normally distributed variables were examined by one-factor analysis of variance (ANOVA), after which post hoc comparison was conducted using Duncan’s multiple range test (*p* ≤ 0.05). Variables with non-normal distributed scores were analysed with non-parametric Kolmogorov–Smirnov tests (*p* ≤ 0.05) and non-parametric Spearman’s Rank-Order Correlation. The parameters that contributed significantly to the final score were determined by stepwise multiple regression analysis. Principal component analysis (PCA) was employed for the simultaneous visualization of the effects of the studied genotypes and traits. All statistical analyses were performed using STATISTICA version 10.0 (Stat Soft Inc., USA, 2011) package.

## Results

### The effectiveness of ME in anther culture

**Fig. 1:**
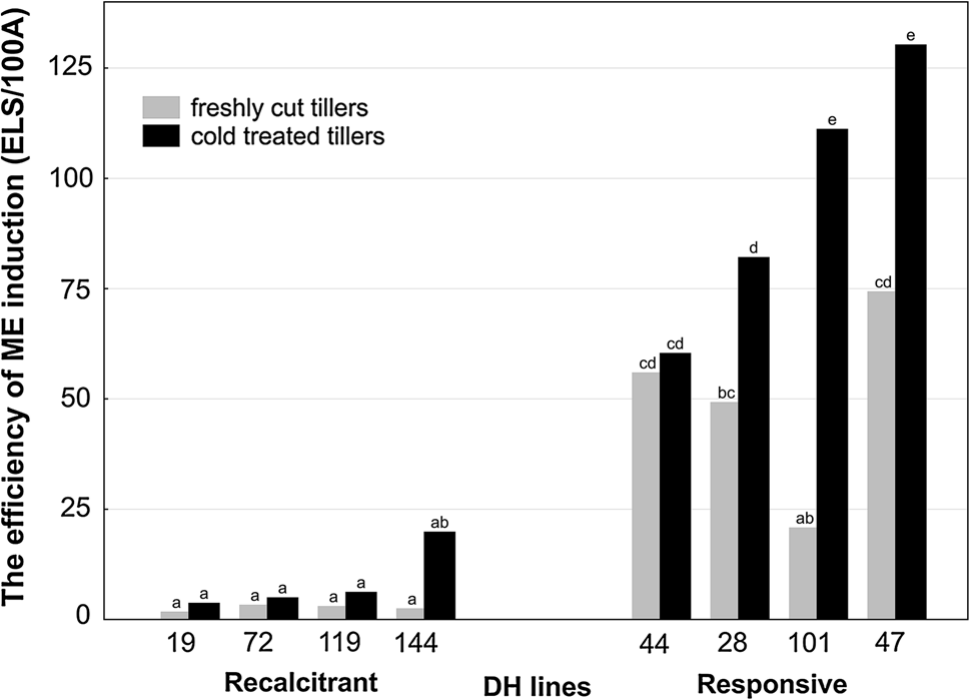
The effect of cold tillers pre-treatment (3 weeks at 4 °C) on the effectiveness of microspore embryogenesis (ME) in anther culture of eight DH lines of winter triticale (× *Triticosecale* Wittm.). Presented data are the means of ten biological replications. Mean values marked with the same *letter* do not differ significantly according to Duncan’s multiple range test (*p* ≤ 0.05). *ELS/100A* the number of embryo-like structures produced per 100 anthers

**Fig. 2:**
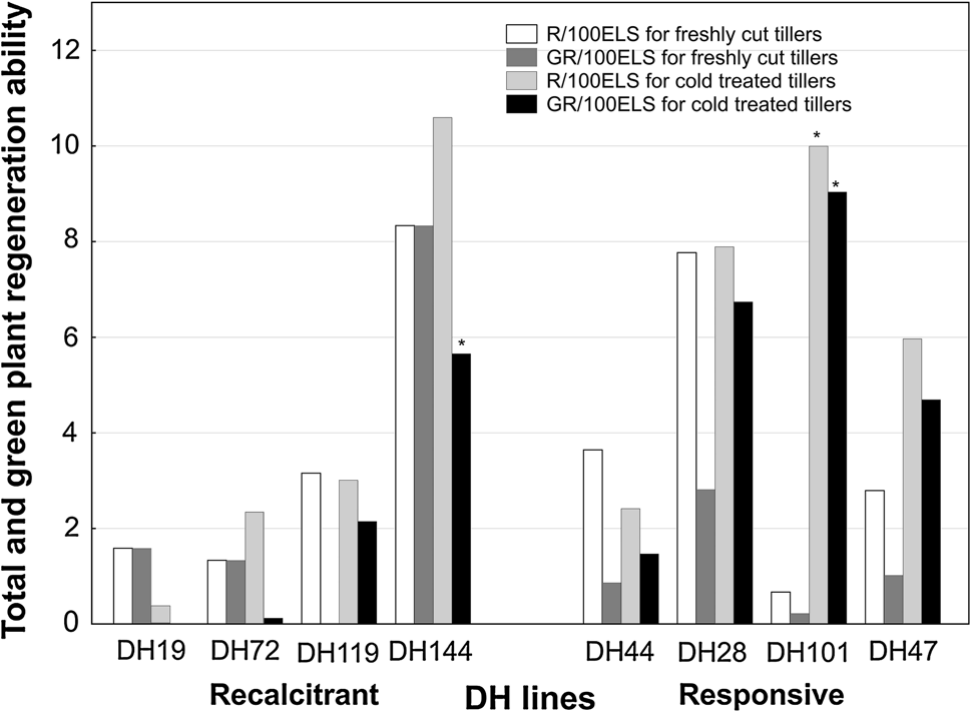
The effect of cold tillers pre-treatment (3 weeks at 4 °C) on the total and green plant regeneration ability in anther culture of eight DH lines of winter triticale (× *Triticosecale* Wittm.). Presented data are the means of ten biological replications. Mean values marked with *asterisk* showed significant difference between the cultures started from freshly cut and cold pre-treated tillers according to Kolmogorov–Smirnov test (*p* ≤ 0.05). *R/100ELS* the number of regenerated plants per 100 embryo-like structures, *GR/100ELS* the number of green regenerated plants per 100 embryo-like structures

The results obtained fully confirmed previous identification of selected DH lines of triticale in respect of their androgenic potential and their classification as ‘responsive’ or ‘recalcitrant’ genotypes. The data for the studied DH lines, ranked according to the increasing effectiveness of ME induction after standard 3-week cold tillers treatment, are presented in Fig. 1. The same ordering of DH lines is continued in all figures presented. Mean effectiveness of ME induction (ELS/100A), both with and without cold treatment, was significantly lower for ‘recalcitrant’ DH lines in comparison with ‘responsive’ ones (Table 1). Significant variation between these two groups was confirmed by non-parametric Kolmogorov–Smirnov test (*p* ≤ 0.05). Cold tillers treatment was not an essential prerequisite for ME induction but significantly increased, almost doubled, the number of produced ELS in ‘responsive’ DH lines (Table 1). In both groups, the produced ELS had similar total regeneration ability (R/100ELS), whereas the effectiveness of green plants production varied depending on the tillers treatment (Fig. 2; Table 1). Generally, regardless of the androgenic potential of the donor plant, ELS derived from non-stressed anthers had similar low ability to regenerate into green plants (Fig. 2). The only exception was DH144 with one of the highest green plant regeneration ability scores. Cold treatment stimulated green plant regeneration but only in the case of ‘responsive’ DH lines, increasing GR/100ELS parameter more than threefold. As a result, ‘responsive’ DH lines were characterized by almost ninefold higher R/100A, and almost threefold higher GR/100A in comparison with ‘recalcitrant’ ones (Table 1).

**Table 1:** The effect of cold treatment (3 weeks at 4 °C) on microspore embryogenesis (ME) induction and plant regeneration ability in triticale (× *Triticosecale* Witm.) anther cultures

Parameter	‘recalcitrant’ DH lines		‘responsive’ DH lines	
	FC	CT	FC	CT
ELS/100A	2.8 ± 0.6	8.5 ± 1.6	50.5 ± 6.6*	98.7 ± 6.7*
R/100ELS	2.5 ± 0.9	4.0 ± 1.2	4.3 ±0.7	6.5 ± 0.8
GR/100ELS	1.2 ± 0.7	1.9 ± 0.7	1.5 ± 0.5	5.4 ± 0.8*
R/100A	0.3 ± 0.1	0.7 ± 0.2	2.9 ± 0.5*	6.1 ± 0.7*
GR/100A	1.2 ± 0.7	1.9 ± 0.7	1.5 ±0.5	5.4± 0.8*

Data are the means (±SE) of four DH lines of triticale identified as ‘recalcitrant’ and ‘responsive’ to ME induction and 10 biological replications for each DH line

Each Petri dish containing 100 anthers from a different spike was assumed to be one biological replication

*ELS/100A* the number of embryo-like structures produced per 100 anthers, *R/100ELS* the number of regenerated plants per 100 embryo-like structures, *GR/100ELS* the number of green regenerated plants per 100 embryo-like structures, *R/100A* the total number of regenerated plants per 100 anthers, *GR/100A* the number of green regenerated plants per 100 anthers

* Significant difference between ‘responsive’ and ‘recalcitrant’ DH lines according to Kolmogorov–Smirnov test (*p* ≤ 0.05)

### Auxs content in triticale anthers and its changes associated with the induction of ME

**Fig. 3:**
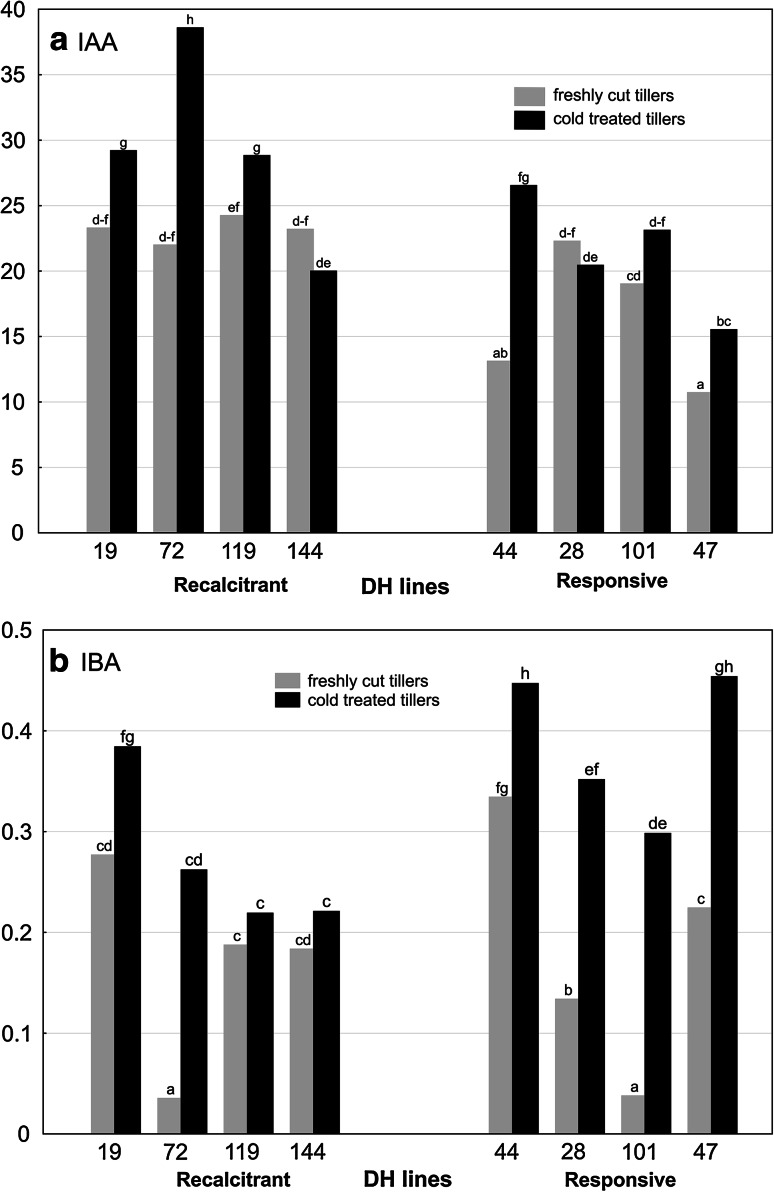
The effect of cold tillers pre-treatment (3 weeks at 4 °C) on **a** IAA and **b** IBA content (nmol g^−1 ^DW) in anthers of eight DH lines of winter triticale (× *Triticosecale* Wittm.). Presented data are the means of six biological replications (samples of anthers collected from different spikes). Mean values marked with the same *letter* do not differ significantly according to Duncan’s multiple range test (*p* ≤ 0.05)

Plant genotype and tillers treatment had a significant effect on Auxs concentration in triticale anthers (Fig. 3a, b; ANOVA analysis, *p* ≤ 0.05). IAA content in anthers isolated from FC tillers varied from 10.8 to 24.3 nmol g^−1^ DW depending on the plant genotype (Fig. 3a). In the same anthers, the concentration of IBA ranged from 0.04 to 0.4 nmol·g^−1^ DW (Fig. 3b), constituting averagely only 1.1 % of total auxin content. Cold treatment (21 days at 4 °C) significantly increased the concentration of both Auxs in anthers of almost all studied DH lines (Fig. 3a, b). Regardless of tillers treatment, significantly lower amount of IAA was detected in anthers of ‘responsive’ DH lines in comparison with ‘recalcitrant’ ones (Table 2). Mean IBA concentration in anthers isolated from FC tillers was similar for both studied groups, whereas after cold tillers treatment anthers of ‘responsive’ genotypes contained significantly higher amount of IBA (Table 2).

**Table 2:** The effect of cold treatment (3 weeks at 4 °C) on plant growth regulators content in triticale (× *Triticosecale* Witm.) anthers

Variable	‘recalcitrant’ DH lines		‘responsive’ DH lines	
	FC	CT	FC	CT
IAA (nmol g^−1^ DW)	23.2^b^	28.7^c^	16.2^a^	21.4^b^
IBA (nmol g^−1^ DW)	0.19^a^	0.27^b^	0.20^a^	0.40^c^
*t*Z (pmol g^−1^ DW)	7.4^c^	3.5^a^	10.5^d^	5.0^b^
*c*Z (pmol g^−1^ DW)	196.8^b^	325.0^c^	155.0^a^	367.6^d^
*t*ZR (pmol g^−1^ DW)	4.6^a^	3.9^a^	5.7^b^	4.1^a^
*c*ZR (pmol g^−1^ DW)	225.5^a^	321.0^b^	212.2^a^	387.4^c^
KIN (pmol g^−1^ DW)	39.6^b^	32.5^b^	34.2^b^	20.1^a^
ABA (nmol g^−1^ DW)	0.90^a^	1.92^b^	0.84^a^	2.98^c^
Auxs Eq/CKs Eq	49.4	42.2	39.3	27.8
Auxs Eq/ABA	26.0	15.1	19.5	7.3
CKs Eq/ABA (×10^3^)	526.6	357.2	497.1	263.2

Data are the means of four DH lines of triticale identified as ‘recalcitrant’ and ‘responsive’ to androgenesis induction and 3–5 biological replications for each DH line

Each Petri dish containing 100 anthers from a different spike was assumed to be one biological replication

Data marked with the same letter do not differ significantly according to the Ducan test (*p* ≤ 0.05)

*IAA* indole-3-acetic acid, *IBA* indolebutyric acid, *tZ* *trans* zeatin, *cZ cis* zeatin, *tZR trans* zeatin riboside, *cZR cis* zeatin riboside, *KIN* kinetin, *ABA* abscisic acid, *Aux Eq* auxin equivalent, the sum of IAA and IBA concentrations, *CKs Eq* cytokinins equivalent, the sum of *t*Z, *c*Z, *t*ZR, *c*ZT and KIN concentrations

### IAA immunolocalization

**Fig. 4:**
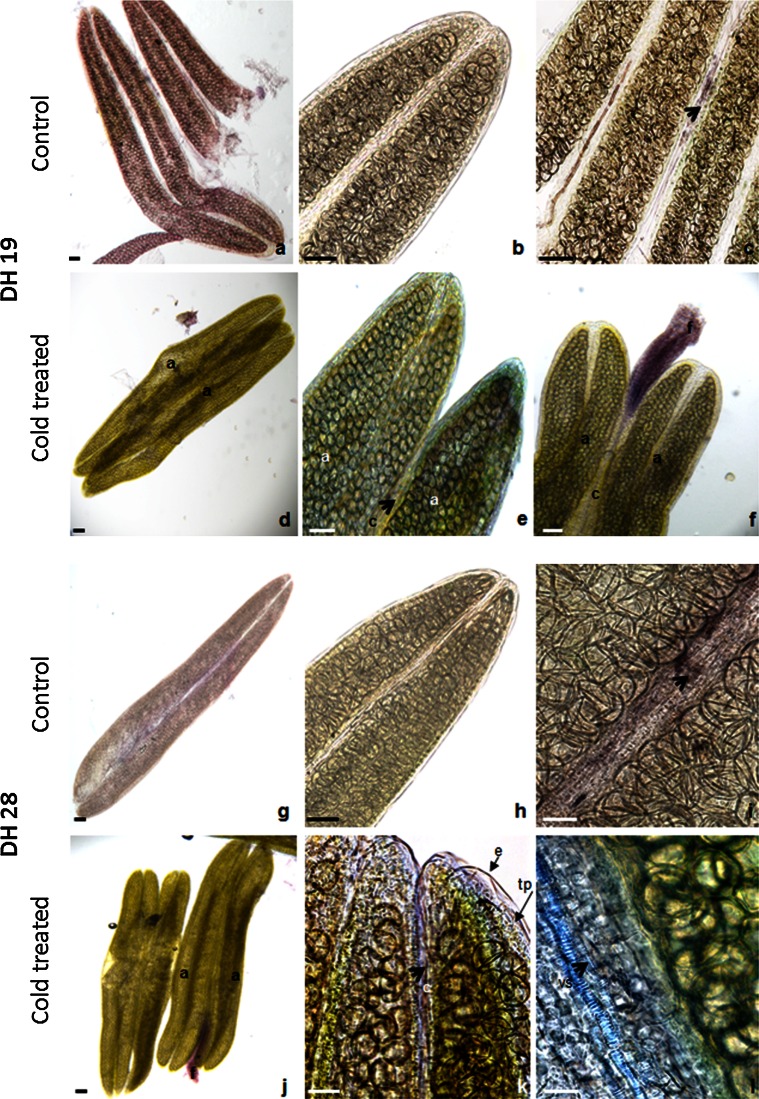
IAA distribution in anthers isolated from ‘recalcitrant’ DH19 (**a**–**f**) and ‘responsive’ DH28 (**g**–**l**) lines. **a–c**, **g–i** Control anthers collected from freshly cut tillers. **d–f**, **j–l** Anthers collected from cold pre-treated tillers. IAA (*violet*) is present in somatic tissue of anther (*a*). Note that the stronger IAA signal occurs in the anther-filament connective (an *arrow* on **c, e**, **i**, **k**), in the protoxylem (px) of the procambial strand (*arrows* on **c**, **l**), in anther outer epidermal cells (*e*) and tapetum (*tp*). *Bar* 20 µm

IAA accumulated in somatic tissue of anthers of both studied DH lines (‘recalcitrant’ DH19 and ‘responsive’ DH28) in the case of FC as well as CT tillers. The most prominent IAA labelling was observed in the anther-filament connective, in the protoxylem of the procambial strand. Moreover, IAA was detectable in tapetum and middle layer cells (Fig. 4).

### CKs content in triticale anthers and its changes associated with the induction of ME

**Fig. 5:**
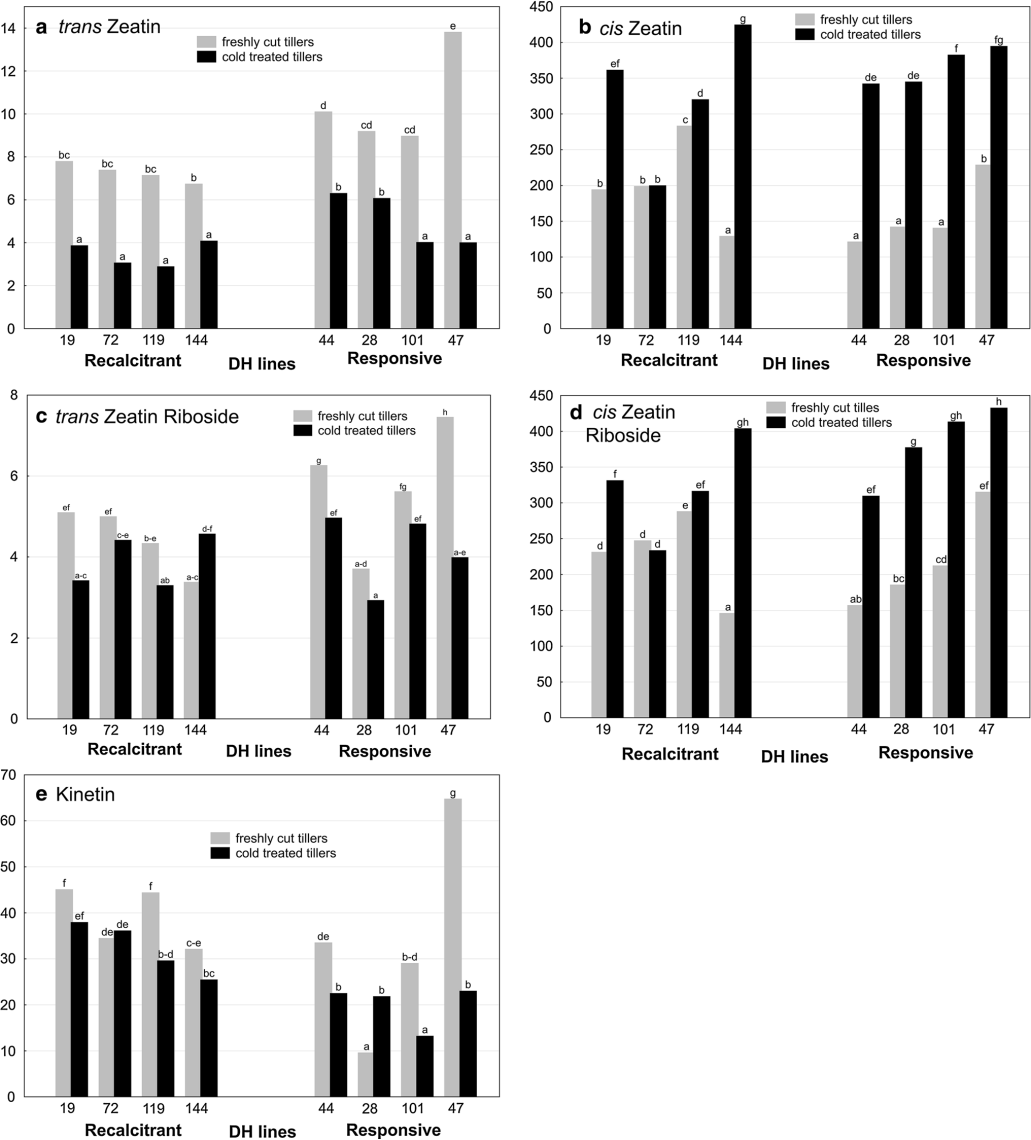
The effect of cold tillers pre-treatment (3 weeks at 4 °C) on the **a** *trans* zeatin, **b** *cis* zeatin, **c** *trans* zeatin riboside, **d** *cis* zeatin riboside and **e** kinetin content (pmol g^−1 ^DW) in anthers of eight DH lines of winter triticale (× *Triticosecale* Wittm.). Presented data are the means of six biological replications (samples of anthers collected from different spikes). Mean values marked with the same *letter* do not differ significantly according to Duncan’s multiple range test (*p* ≤ 0.05)

*Trans* and *cis* isomers of zeatin (*t*Z, *c*Z) and zeatin riboside (*t*ZR, *c*ZR) as well as kinetin (KIN) were detected in anthers of all studied DH lines (Fig. 5a–e). *Cis* isomers prevailed significantly, by two orders of magnitude, both in free (with the mean of 254.1 vs. 6.7 pmol g^−1^ DW) and conjugated forms (280.9 vs. 4.6 pmol g^−1^ DW) of zeatin. In this respect, KIN concentration with the mean of 32 pmol g^−1^ DW can be considered as moderate. To confirm the obtained results, the peak of KIN in the samples was identified in several ways. First, it was compared to external standard retention time (online resource 1). In all cases, the peak identified as KIN had retention time of about 18 min. Mass spectrograph was set to MRM (multiple reactions monitor) mode, ion 81.2 produced from ion 220.2 (marked as 220.2→81.2) was chosen for quantification. Two additional ions (220.2→148.3 and 220.2→188.3) were used to increase the proof of identification. Mass spectra with abundance of these ions were compared for all samples with the peak identified as KIN (online resource 2). All samples were spiked with internal isotope standard—[^15^N_4_] KIN (all atoms of nitrogen substituted with higher isotope ^15^N)—to monitor any changes in retention time. The stability and purity of this internal standard was additionally checked and no presence of KIN with all four atoms ^14^N was found; therefore, the identification of internal standard impurities as natural KIN in the samples was not possible.

Although CKs concentration was under genomic control, it was significantly influenced by cold tillers treatment (ANOVA analysis, *p* ≤ 0.05). The data for each DH line are presented in Fig. 5a–e. Low temperature treatment induced intense accumulation of *c*Z (Table 2), with 1.6- and 2.4-fold increase of its concentration in anthers of ‘recalcitrant’ and ‘responsive’ DH lines, respectively. Similar increase in *c*ZR concentration was detected in CT anthers of both ‘responsive’ and ‘recalcitrant’ DH lines (Table 2). In contrast, regardless of androgenic potential, the concentration of *t*Z diminished twofold, whereas *t*ZR content remained unchanged in anthers of ‘recalcitrant’ DH lines and decreased by almost 30 % in anthers of ‘responsive’ genotypes. The concentration of KIN in anthers isolated from FC tillers was genotype dependent and not connected with androgenic potential of the donor plant (Fig. 5e; Table 2). In the majority of ‘responsive’ DH lines, a significant decrease of KIN concentration in response to low temperature was detected.

In summary, ‘responsive’ DH lines were characterized by significantly higher *t*Z, *t*ZR and lower *c*Z level in anthers isolated from FC tillers as compared with ‘recalcitrant’ DH lines (Table 2). After cold tillers treatment, higher content of *t*Z, *c*Z, *c*ZR and lower concentration of KIN were the parameters that distinguished ‘responsive’ from ‘recalcitrant’ DH lines.

### ABA content in triticale anthers and its changes associated with the induction of ME

**Fig. 6:**
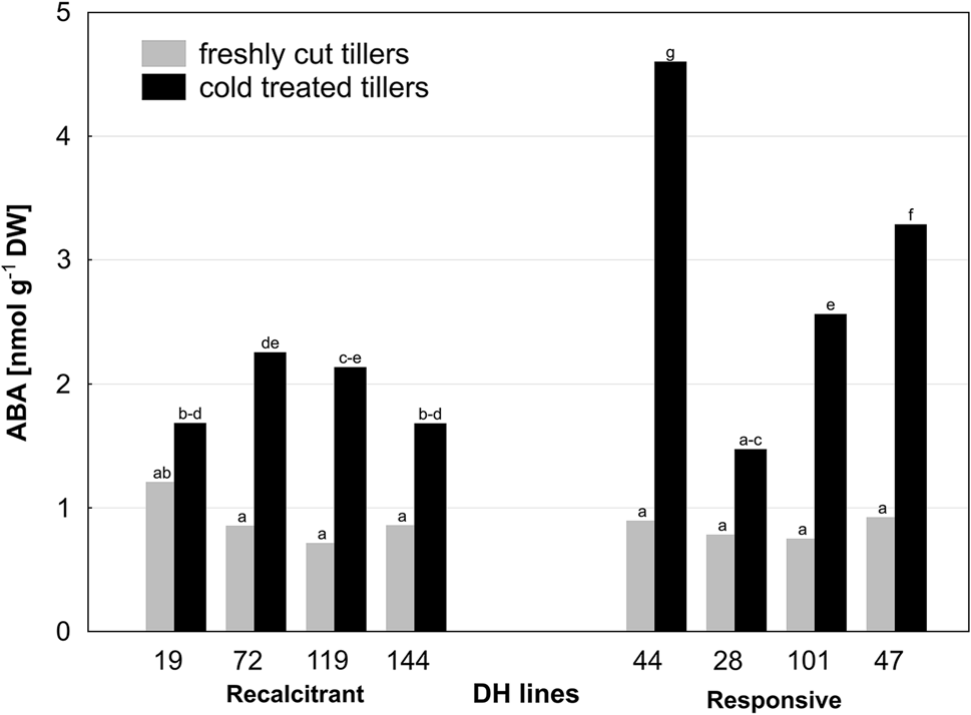
The effect of cold tillers pre-treatment (3 weeks at 4 °C) on ABA content in anthers of eight DH lines of winter triticale (× *Triticosecale* Wittm.). Presented data are the means of six biological replications (samples of anthers collected from different spikes). Mean values marked with the same *letter* do not differ significantly according to Duncan’s multiple range test (*p* ≤ 0.05)

The concentration of ABA in anthers of the studied DH lines of triticale varied from 0.66 to 1.21 nmol g^−1^ DW (Fig. 6). Like in the case of the other PGRs, plant genotype and low temperature treatment had a significant effect on ABA concentration (ANOVA analysis, *p* ≤ 0.05). Endogenous content of ABA was approximately at the same level in FC anthers of ‘recalcitrant’ and ‘responsive’ DH lines (Table 2). Intensive ABA accumulation (with a mean threefold increase) was observed in response to cold tillers treatment in anthers of all studied DH lines (Fig. 6). However, the increase in ABA concentration was significantly higher in ‘responsive’ DH lines (Table 2).

### The effect of altered hormonal balance on triticale ME

In anther cultures of ‘recalcitrant’ DH19 line, none of the modifications of tillers treatment had a significant effect on the effectiveness of ME induction (ELS/100A). However, it could be seen that both short- and long-term treatment with 10 µM ABA slightly stimulated ELS formation (Table 3). This effect was associated with 1.5–2.5-fold increase in endogenous ABA content in comparison with non-treated plants (Table 4). The only significant effect on ELS regeneration ability was induced by adding 5 µM PCIB to Hoagland’s salt solution during cold tillers treatment, 3 days before anthers isolation. PCIB stimulated total regeneration ability, but its effect on green plant production was not significant (Table 3). Endogenous IAA concentration in PCIB-treated anthers decreased twofold, which was associated with 2.5-fold increase of IBA content (Table 5). However, even this positive PCIB influence did not affect final ME effectiveness, which for this ‘recalcitrant’ DH line varied between 0 and 0.6 GR/100A.

**Table 3:** The effect of exogenous ABA (10 µM exABA), ABA biosynthesis inhibitor (5 µM NOR), inhibitor of auxin transport (10 µM TIBA) and anti-auxin (5 µM PCIB) on the effectiveness of microspore embryogenesis in anther cultures of two DH lines of triticale (× *Triticosecale* Wittm.) with significantly different androgenic responsiveness (‘responsive’ DH28 and ‘recalcitrant’ DH19)

Treatment	Days of treatment	ELS/100A		R/100ELS		GR/100ELS		GR/100A	
		DH19	DH28	DH19	DH28	DH19	DH28	DH19	DH28
Control*	3	3.1 ± 2.0	122.8 ± 19.8	8.3 ± 8.3	13.6 ± 3.4	8.3 ± 8.3	12.1 ± 3.0	0.6 ± 0.6	14.4 ± 3.7
	21	1.1 ± 0.7	97.6 ± 10.4	0	16.2 ± 3.1	0	15.4 ± 3.0	0	13.9 ± 2.6
exABA	3	7.7 ± 2.8	**57.9 ± 20.1**	6.7 ± 6.7	6.0 ± 2.3	0	6.0 ± 2.3	0	**4.6 ± 2.6**
	21	5.5 ± 1.8	98.5 ± 7.8	11.0 ± 6.8	8.2 ± 1.2	10.4 ± 6.9	**7.2 ± 1.0**	0.3 ± 0.1	7.5 ± 1.5
NOR	3	4.7 ± 2.4	105.8 ± 16.3	2.0 ± 2.0	8.4 ± 1.6	2.0 ± 2.0	7.8 ± 1.2	0.2 ± 0.2	8.1 ± 1.6
	21	5.2 ± 1.2	109.4 ± 11.7	7.1 ± 3.9	**5.6 ± 1.1**	6.6 ± 4.0	**4.9 ± 1.1**	0.4 ± 0.2	**5.3 ± 1.3**
TIBA	3	2.5 ± 1.4	127.3	8.3 ± 8.3	11.3	8.3 ± 8.3	8.7	0.4	12.9
	21	4.2 ± 0.9	117.9 ± 10.2	11.7 ± 5.7	10.7 ± 1.2	6.5 ± 3.7	9.7 ± 1.2	0.3 ± 0.2	10.7 ± 1.4
PCIB	3	4.0 ± 2.0	116.7 ± 17.6	**15.0 ± 12.4**	12.6 ± 5.2	15.0 ± 12.4	12.6 ± 5.2	0.6 ± 0.4	16.7 ± 10.0
	21	2.4 ± 1.1	102.9 ± 14.8	4.7 ± 3.9	10.0 ± 2.4	0	9.1 ± 2.2	0	8.6 ± 1.8
DMSO	3	5.9 ± 2.4	112.2 ± 13.3	0	**4.6 ± 1.4**	0	4.6 ± 1.4	0	**5.6 ± 2.0**
	21	4.8 ± 1.0	81.2 ± 7.3	12.7 ± 4.6	11.4 ± 0.7	9.1 ± 4.3	10.9 ± 0.9	0.4 ± 0.2	8.8 ± 0.9

All compounds were supplemented to Hogland’s medium at the start of cold tillers treatment (21 days at 4 °C) or 3 days before anthers isolation. DMSO (PCIB solvent) in 0.1 % concentration was used as additional control

Data are the means of 5–7 biological replications ±SE

Each Petri dish containing 100 anthers from a different spike was assumed to be one biological replication

Statistical analysis separate for each parameter and treatment; data marked in *bold* differ significantly in comparison with control according to non-parametric Kolmogorov–Smirnov test (K–S), *p* ≤ 0.05

Final efficiency of androgenesis was estimated by GR/100A—the number of green regenerants per 100 isolated anthers

*ELS/100A* the number of embryo-like structures produced per 100 anthers of the donor plant, *R/100ELS* total number of regenerants per 100 embryo-like structures transferred to the regeneration medium, *GR/100ELS* the number of green regenerants per 100 embryo-like structures transferred to regeneration medium, *ABA* abscisic acid, *NOR* norflurazone, *TIBA* 2,3,5-triiodobenzoic acid, *PCIB* p-chlorophenoxyisobutyric acid

* Cultures of anthers excised from tillers kept during whole cold treatment in Hoagland’s medium (21 days of treatment) or transferred 3 days before anthers isolation to fresh Hoagland’s medium (3 days of treatment)

**Table 4:** The effect of exogenous abscisic acid (10 µM exABA) and ABA biosynthesis inhibitor (5 µM NOR) tillers treatments on endogenous level of ABA in anthers of two DH lines of triticale (× *Triticosecale* Wittm.) with significantly different androgenic responsiveness (‘responsive’ DH28 and ‘recalcitrant’ DH19)

Treatment	Days of treatment	ABA (nmol g^−1^ DW)	
		DH19	DH28
Control*		1.7 ± 0.2	1.9 ± 0.1
exABA	3	2.3 ± 0.3	2.6 ± 0.2
	21	4.3 ± 0.5	2.2 ± 0.1
NOR	3	1.5 ± 0.1	1.9 ± 0.2
	21	2.2 ± 0.2	2.2 ± 0.2

Both compounds were supplemented to Hogland’s medium at the start of cold tillers treatment (21 days at 4 °C) or 3 days before anthers isolation

Data are the means of 3 biological replications ±SE

Sample of anthers collected from several spikes with total fresh weight of 0.3 g was assumed to be one biological replication

*ex ABA* exogenous abscisic acid, *NOR* norflurazone

* Anthers excised from tillers kept during the whole period of cold treatment (21 days) in Hoagland’s medium

**Table 5:** The effect of auxin transport inhibitor (10 µM TIBA) and anti-auxin (5 µM PCIB) tillers treatment on endogenous level of IAA and IBA in anthers of two DH lines of triticale (× *Triticosecale* Wittm.) with significantly different androgenic responsiveness (‘responsive’ DH28 and ‘recalcitrant’ DH19)

Treatment	Days of treatment	IAA (nmol g^−1^ DW)		IBA (nmol g^−1^ DW)	
		DH19	DH28	DH19	DH28
Control*		30.5 ± 4.1	19.2 ± 0.8	0.38 ± 0.03	0.33 ± 0.01
TIBA	3	41.1 ± 4.8	26.7 ± 3.1	0.31 ± 0.01	0.38 ± 0.04
	21	14.6 ± 7.7	6.0 ± 0.2	2.1 ± 0.31	0.43 ± 0.02
PCIB	3	14.1 ± 1.5	23.8 ± 2.8	0.96 ± 0.20	0.33 ± 0.04
	21	27.7 ± 3.1	41.8 ± 6.0	0.53 ± 0.13	0.33 ± 0.01
DMSO	3	19.1 ± 0.8	42.5 ± 4.0	1.02 ± 0.01	0.36 ± 0.01
	21	46.6 ± 13.9	26.7 ± 8.5	0.40 ± 0.02	0.39 ± 0.01

DMSO (PCIB solvent) in 0.1 % concentration was used as additional control. All compounds were supplemented to Hogland’s medium at the start of cold tillers treatment (21 days at 4 °C) or 3 days before anthers isolation

Data are the means of 3 biological replications ±SE

Sample of anthers collected from several spikes with total fresh weight of 0.3 g was assumed to be one biological replication

*TIBA* 2,3,5-triiodobenzoic acid, *PCIB* p-chlorophenoxyisobutyric acid, *DMSO* dimethyl sulfoxide

* Anthers excised from tillers kept during the whole cold treatment (21 days) in Hoagland’s medium

In contrast, in highly ‘responsive’ DH28, short-term ABA treatment significantly decreased ME induction (Table 3). The effectiveness of the process diminished more than twofold in cultures of anthers isolated from 3-day ABA-treated tillers. This effect was associated with 35 % increase of endogenous ABA content in the anthers of ABA-treated DH28 tillers (Table 4). ABA applied at the start of cold treatment (21 days at 4 °C) increased its endogenous concentration by 16 % and also had a negative effect on green plant regeneration ability (Table 3). Even more pronounced negative effect was induced by long-term 5 µM NOR treatment, inhibitor of ABA biosynthesis. Though endogenous ABA content in anthers isolated from long-term NOR-treated plants did not change significantly, it decreased both total and green plant regeneration ability. Negative effect on total plant regeneration ability was also induced by 0.1 % DMSO, used as the control for PCIB treatment.

The effectiveness of ME induction was not improved by any modification of the induction medium C17 (Table 6). The supplementation of TIBA and PCIB did not change ME induction effectiveness (ELS/100A) in the case of ‘recalcitrant’ DH19 and significantly decreased this parameter in anther cultures of ‘responsive’ DH28. Moreover, deprivation of exogenous synthetic Auxs analogues from C17 medium, which standardly contains 1 mg l^−3^ Dicamba, 1 mg l^−3^ Picloram and 0.5 mg l^−3^ KIN, resulted in drastic inhibition of ELS development, decreasing the number of produced ELS from 91 (control) to 57 (TIBA), 67 (PCIB) and 11 (noAuxs) per 100A. Although the tested substances had no effect on ELS regeneration ability, final androgenesis effectiveness decreased significantly from 22 (control) to 14 (TIBA) and 3 (noAuxs) GR/100A.

**Table 6:** The effect of inhibitor of auxin transport (10 µM TIBA) and anti-auxin (5 µM PCIB) supplemented to C17 induction medium and auxin deprivation from C17 (noAuxs C17) on the effectiveness of microspore embryogenesis in anther cultures of two DH lines of triticale (× *Triticosecale* Wittm.) with significantly different androgenic responsiveness (‘responsive’ DH28 and ‘recalcitrant’ DH19)

Treatment	ELS/100A		R/100ELS		GR/100ELS		GR/100A	
	DH19	DH28	DH19	DH28	DH19	DH28	DH19	DH28
Control C17*	3.8 ± 2.1	91.0 ± 13.3	12.5 ± 12.5	42.5 ± 10.2	0	25.8 ± 8.4	0	21.6 ± 5.8
C17+TIBA	3.2 ± 1.5	**56.8 ± 4.5**	32.7 ± 14.6	43.8 ± 3.9	8.0 ± 8.0	23.7 ± 2.3	0.6 ± 0.6	13.8 ± 2.4
C17+PCIB	2.3 ± 0.7	**67.4 ± 10.0**	39.2 ± 15.8	43.8 ± 3.0	15.0 ± 9.5	26.3 ± 2.5	0.4 ± 0.3	17.1 ± 1.7
C17+DMSO	1.7 ± 0.8	82.1 ± 7.1	52.1 ± 16.8	30.5 ± 4.0	25.0 ± 25.0	21.0 ± 4.4	0.2 ± 0.2	16.0 ± 2.9
noAuxs C17	0	**10.8 ± 2.8**	–	51.1 ± 13.1	–	23.9 ± 11.6	–	3.3 ± 1.8

Data are the means of 5–7 biological replications ±SE

Each Petri dish containing 100 anthers from a different spike was assumed to be one biological replication

Statistical analysis separate for each parameter and treatment

Data marked in *bold* differ significantly in comparison with the control; according to non-parametric Kolmogorov–Smirnov test (K–S), *p* ≤ 0.05

Final efficiency of androgenesis was estimated by GR/100A—the number of green regenerants per 100 isolated anthers

*ELS/100A* the number of embryo-like structures produced per 100 anthers of the donor plant, *R/100ELS* total number of regenerants per 100 embryo-like structures transferred to the regeneration medium, *GR/100ELS* the number of green regenerants per 100 embryo-like structures transferred to regeneration medium

* C17 induction medium containing 1 mg dm^−3^ Dicamba, 1 mg dm^−3^ Picloram and 0.5 mg dm^−3^ kinetin

### Identification of parameters associated with high ME responsiveness by statistical approaches

In the case of cultures started from FC tillers, non-parametric Spearman’s Correlation test detected significant relationship between the effectiveness of ME induction (ELS/100A) and IAA (*R* = −0.86) as well as *t*Z (*R* = 0.86) content in triticale anthers. None of the tested variables significantly correlated with regeneration ability of produced ELS. Final ME effectiveness could be predicted by measuring concentrations of several PGRs, as it is shown by multiple regression equation: GR/100A = 4.8 + 0.03 *c*Z + 0.7 × *t*Z* − 2.3 × *t*ZR* − 1.8 × IAA* + 0.4 × ABA* − 0.2 × IBA; where (*) means statistical significance at *p* ≤ 0.05.

The value of *B* coefficient indicates that *t*Z and ABA had significant stimulating effects, whereas high level of *t*ZR and IAA decreased the effectiveness of the process. The effect of these variables contributed 99 % of the variation in the final effectiveness of ME.

In cultures of anthers excised from CT tillers, strong negative correlation was detected between ME induction (ELS/100A) and content of both IAA (*R* = −0.81) and KIN (*R* = −0.86), whereas positive effect was induced by *c*ZR (*R* = 0.71). High concentration of IAA diminished also the regeneration ability of produced ELS (*R* = −0.79 for R/100ELS and −0.71 for GR/100ELS). The effect of KIN was less detrimental as it was negatively correlated only with green plant regeneration ability (*R* = −0.83 GR/100ELS). Multiple regression analysis revealed that the tested variables explained 94 % of the variation in final ME effectiveness and only Z and *cis* isomer ZR showed significant contribution: GR/100A = −30 + 2.5×*c*ZR* − 1.4 × *c*Z* + 0.6 × *t*Z* + 0.7×IAA. Among them, only *c*Z showed a negative influence (negative coefficient B).

**Fig. 7:**
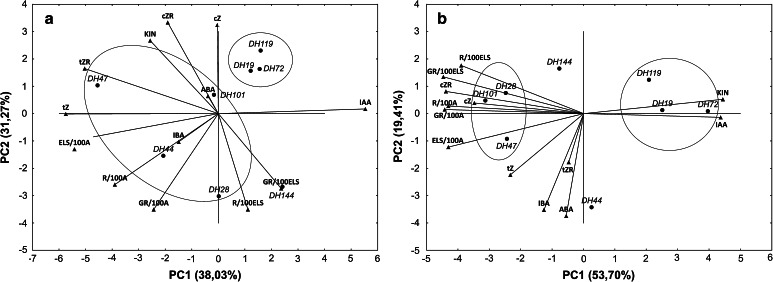
Vector view of genotype-by-trait principal component analysis (PCA) showing interrelationships among studied variables in anthers excised from freshly cut (**a**) and cold pre-treated tillers (**b**). *ELS/100A* the number of embryo-like structures produced per 100 anthers, *R/100ELS* the number of regenerated plants per 100 embryo-like structures, *GR/100ELS* the number of green regenerated plants per 100 embryo-like structures, *R/100A* the total number of regenerated plants per 100 anthers, *GR/100A* the number of green regenerated plants per 100 anthers

Also, PCA was used separately for various tillers treatments (FC, CT; Fig. 7a, b) to visualize similarities of the interrelationships among studied traits and genotypes. The analyses based on the first two principal components (PC) explained a high proportion of the total variation (69 and 73 % for FC and CT, respectively) and indicated a significant effect of cold tillers treatment on the relation pattern among tested variables.

For FC tillers, the first PC (38 % of the total variation) was associated with ME induction effectiveness (ELS/100A) and confirmed its negative correlation with IAA as indicated by the obtuse angle between their vectors (Fig. 7a). According to this analysis, ME induction was positively correlated not only with concentrations of *t*Z but also with *t*ZR. The second PC (31.3 % of the total variation) was strongly associated with regeneration ability (R/100ELS, GR/100ELS) and final ME effectiveness (GR/100A), and showed strong negative correlation with *cis* isomers of Z and ZR as well as KIN. It could be seen that values of PC1 strongly discriminated ‘responsive’ genotypes from ‘recalcitrant’ ones. Three among four ‘recalcitrant’ DH lines of triticale were quite similar in their response, whereas DH144 presented a different pattern. Such uniformity was not typical for ‘responsive’ DH lines.

After cold tillers treatment (CT), all parameters describing ME effectiveness and regeneration ability were associated in PC1 (53.7 % of the total variation; Fig. 7b). The analysis confirmed strong negative correlations between all parameters describing the process of ME (ELS/100A; R/100ELS, GR/100ELS; R/100A; GR/100A) and both IAA and KIN. According to this analysis, ELS/100A was positively correlated not only with *c*ZR but also with *c*Z. Again three among four ‘recalcitrant’ DH lines responded quite uniformly, with the exception of DH144. The same situation could be observed in the group of ‘responsive’ genotypes, with DH44 characterized by a divergent response pattern.

## Discussion

Various kinds of stress factors applied separately or in combinations can induce efficient microspore reprogramming, but the precise mechanism of this process is still unclear. In triticale, long-term low temperature tillers pre-treatment (2–3 weeks at 4 °C) has turned out to be the most efficient factor in ME induction and is standardly used in various lab protocols (Pauk et al. 2003; Wędzony 2003; Żur et al. 2009). Low temperature affects many physiological processes in plant cells, slows down growth rate and degradation, affects cytoskeleton, activates Ca^2+^ pathways, stimulates expression of small heat shock proteins (Touraev et al. 1997; Zoriniants et al. 2005) and affects the activity of antioxidative system (Żur et al. 2014). Since one of the most dynamic changes noticed under stress conditions is perturbed PGRs homeostasis (Kohli et al. 2013), it seems obvious that it is also associated with stress-induced ME (Dollmantel and Reinert 1980; Gorbunova et al. 2001; Hays et al. 2001; Dubas et al. 2013a, b). In the presented study, the changes in Auxs, CKs and ABA concentration in anthers in response to ME-inducing treatment (21 days at 4 °C) were monitored and compared among eight DH lines of triticale differing in androgenesis responsiveness, giving us new information about hormonal balance determining effective embryogenic development of microspores.

A certain concentration of IAA is necessary for normal development of anthers—its lack could cause pollen abortion and male sterility (Tang et al. 2008). In *Arabidopsis*, Auxs regulate early stages of stamen development (Cheng et al. 2006) and according to recent reports coordinate also anther dehiscence, pollen development, and pre-anthesis filament elongation (Cecchetti et al. 2008). Relatively high level of free IAA, about 5 nmol g^−1^ FW, which was 64 times higher in comparison with the leaf blade level, was detected in the anthers of rice suggesting that this PGR plays an important role in anther development (Hirano et al. 2008). This result is very similar to data obtained in our study (Fig. 3), where depending on the plant genotype and tillers treatment, the concentration of IAA in triticale anthers ranged from 10.7 to 38.6 nmol g^−1^ DW. Surprisingly, higher IAA concentration in anthers excised from FC (by 43 %) as well as CT tillers (by 34 %) was a conspicuous feature that characterized ‘recalcitrant’ DH lines. This observation was confirmed by the results of correlation analysis indicating a strong negative correlation between IAA concentration and the effectiveness of ME induction (ELS/100A). Similar results were reported by Gorbunova et al. (2001), who suggested that the proper balance between the amount of endogenous and exogenous Auxs provided by the induction medium is essential for effective ME induction.

To widen our knowledge in respect of cellular localization and transport of IAA, a ‘whole-mount’ cytochemistry protocol using an anti-IAA-monoclonal antibody was established. IAA was detected in somatic tissues of anthers of both analysed DH lines of triticale, regardless of tillers treatment (Fig. 4). IAA localization in procambial strands of anther-filament connective, in tapetum and middle layer cells, corresponds to the auxin reporter *DR5::β*-*glucuronidase* (*GUS*) activity in *Arabidopsis* (Cecchetti et al. 2008). It could be supposed that IAA-labelled sites such as tapetum and middle layer cells are the local auxin sources, whereas the presence of IAA in procambial cells denotes auxin transport through the filament to an anther (Cecchetti et al. 2008). Other reports also suggested that early phases of anther development are based on local IAA biosynthesis mainly in the tapetum cells which then supply this Aux to pollen grains (Aloni et al. 2006; Cecchetti et al. 2008). In contrast to IAA, higher concentration of IBA seems to be advantageous for effective ME induction (Table 2). IBA is an endogenous Aux which has been detected in various plant species but the studies on its role are rather limited. According to the majority of data, the activity of this Aux seems to result from its conversion to IAA in the process of peroxisomal β-oxidation (Woodward and Bartel 2005; Baker et al. 2006). However, some evidence suggests that it may also act directly, not only as IAA precursor (Ludwig-Müller 2000; Poupart and Waddell 2000; Zolman et al. 2000). As the activity of IBA synthase, using IAA as a substrate for IBA production, is induced by ABA (Ludwig-Müller et al. 1995), it seems probable that this pathway took place also in triticale anthers. However, because IBA pool in triticale anthers comprises only about 1 % of the total Auxs content, it is questionable whether changes in its concentration are of any significance. A possible role of IBA in *Brassica napus* microspore embryogenesis has been postulated by Dubas et al. (2013b), but this assumption needs to be further substantiated.

Drastic decrease of ELS formation as the effect of total deprivation of synthetic Auxs from C17 medium and negative effects of TIBA and PCIB supplementation observed in anther cultures of ‘responsive’ DH28 line (Table 6) confirms an earlier hypothesis (Gorbunova et al. 2001) that proper balance between endogenous and exogenous Auxs is important for efficient ME. It seems to be established in anther cultures of ‘responsive’ DH28 line and any disturbance induced by chemical treatment could only diminish the effectiveness of the process.

Positive effect of short-term PCIB tillers treatment on ELS regeneration ability of ‘recalcitrant’ DH19 line (Table 3) suggests that high endogenous IAA level together with exogenous Auxs supplemented to the culture media suppresses plant regeneration. Due to structural similarity, PCIB can compete with IAA at the binding site of its receptor and thus overcome the inhibitory effect of high endogenous IAA concentration (Hadfi et al. 1998; Michalczuk et al. 1992; Oono et al. 2003; Agarwal et al. 2006). Among others, PCIB promoted the development of somatic embryos of *Abies normandiana* (Find et al. 2002) as well as the development of zygotic and microspore-derived embryos of *Brassica juncea* (Hadfi et al. 1998; Agarwal et al. 2006). On the other hand, removal of all exogenous Aux from the induction medium completely blocked ELS formation (Table 6), which again confirms that certain, probably genotype-dependent, endogenous/exogenous Auxs balance is important for effective ME induction. The obtained results did not confirm the beneficial effect of IAA polar transport inhibitor (TIBA) on anther culture responses which had been observed in maize and barley (Pretova et al. 1993; Cistué et al. 1998). Similar results received by Choi et al. (1997, 2001) and Venkatesh et al. (2009) suggest that also non-zygotic in vitro embryo formation requires the establishment of IAA gradient resulting from polar transport.

The level of CKs detected in triticale anthers (2.9–13.8 pmol g^−1^ DW; Fig. 5) is in agreement with data from rice anthers analysis (Hirano et al. 2008). In rice, the level of *t*Z and its precursor *t*ZR did not exceed 2.5 pmol g^−1^ FW and such small accumulation gave the authors reason to conclude that this type of PGR is not important for anther development. However, in the case of triticale anthers, *cis* isomers of Z and ZR turned out to be the prevailing forms of CK, detected in concentrations manifold higher than *trans* isomers (Fig. 5). Based on studies with *Arabidopsis*, *cis* isomers were regarded as CK derivatives without any or with low biological activity. Further studies with non-model plants showed that *cis* isomers can be the dominant form of CK in specific plant organs and/or stages of development (Emery et al. 1998; Vyroubalová et al. 2009; Kudo et al. 2012). In recently published report, Gajdošová et al. (2011) proposed that *c*Z can be qualified as a regulator of CK responses in plants under growth-limiting conditions. The involvement of *c*Z in ME seems to be confirmed by the results of PCA (Fig. 7), which detected positive correlation between *c*ZR, *c*Z and the induction of ME (ELS/100A) as well as the regeneration ability of produced ELS (R/100ELS, GR/100ELS) in cold-stressed anther cultures. Without cold stress, ME induction was positively correlated with *t*Z (*R* = 0.86) and *t*ZR (*R* = 0.60), whereas *cis* isomers negatively correlated with regeneration parameters (R/100ELS, GR/100ELS). Although cold stress treatment surely creates growth-limiting conditions, physiological significance of *cis* CK isomers remains unclear and their involvement in the process of ME needs further clarification.

For a long time, KIN has been regarded as a synthetic product of DNA rearrangement and only recently it was detected as native PGR in some plant species (reviewed in Barciszewski et al. 1999, 2007; Ge et al. 2005). Relatively high level of KIN (10–64 pmol g^−1^ DW) detected in triticale anthers (Fig. 5) is rather surprising, but high standards of the analysis together with appropriate precautions taken guarantee that this result is not a methodological artefact. It can be the result of organ specificity as anthers differ significantly in their hormonal requirements from other tissues of the maternal plant (Gorbunova et al. 2001; Hirano et al. 2008). Some data indicated that high levels of KIN can induce the process of programmed cell death (PCD), e.g., in root cortex cells of *Vicia faba* (review in Kunikowska et al. 2013). On the other hand, strong antioxidant properties of KIN and some ABA-antagonistic effects suggest that KIN functions as an anti-stress agent by preventing formation of reactive oxygen species (ROS). It is possible that high concentration of KIN in cold-stressed anthers of ‘recalcitrant’ DH lines, which correlated negatively with the effectiveness of ME, reflects higher ROS generation, which has been revealed in another study (Żur et al. 2014).

Among various PGRs, ABA is the most thoroughly studied hormone for plant stress response. Its accumulation under stress is a common reaction in the plant kingdom associated with the initiation of plant defence mechanisms and usually positively correlated with the level of stress tolerance (Christmann et al. 2004; Wilkinson and Davies 2002). In anthers, ABA prevents premature development and, under specific circumstances, initiates pollen abortion. Relatively low level of ABA is characteristic for normally developing anthers (Tang et al. 2008). Several reports suggested ABA involvement in androgenesis induction and documented its positive effect on the effectiveness of the process (Imamura and Harada 1980; Reynolds and Crawford 1996; Van Bergen et al. 1999; Wang et al. 1999; Guzman and Arias 2000). Data published recently (Żur et al. 2012) showed intensive ABA accumulation in cold-treated anthers of triticale. However, no correlation between ABA concentration and ME induction effectiveness was detected. This result was confirmed in this study, as although ‘responsive’ DH lines accumulated significantly higher amount of ABA in response to cold tillers treatment (Fig. 6), no correlation between ABA concentration and ME effectiveness was found. It seems that ME induction requires a certain genotype-specific threshold level of ABA but too high accumulation diminishes the effectiveness of the process. This hypothesis seems to be confirmed by the results of the experiment in which cold stress was combined with short-term exogenous ABA tillers treatment. Additional 30 % increase of endogenous ABA concentration (Table 4) decreased final ME effectiveness of highly ‘responsive’ DH28 line more than threefold (Table 3). The observed negative effect of ABA biosynthesis inhibitor (NOR) on both total and green plant regeneration ability seems to be unconnected with ABA effect as no significant change in endogenous level of ABA under NOR treatment was detected.

## Conclusions

In conclusion, the results presented suggest concerted and complex Auxs/CKs/ABA crosstalk in the regulation of cold stress-induced ME in anther culture of triticale. Even if several distinct features discriminate highly embryogenic systems from less effective or highly ‘recalcitrant’ ones (Table 2), it seems that none of them acts alone in the acquisition of embryogenic competency. Thus, it seems that the most important prerequisite for effective ME is a specific PGRs homeostasis. Highly efficient system (‘responsive’ DH lines after cold tillers treatment; Table 2) was quite specific in respect of hormonal balance between Aux equivalent (Aux Eq = IAA+IBA), CK equivalent (CK Eq = *c*Z + *t*Z + *c*ZR + *t*ZR + KIN) and ABA. All ratios calculated (Aux Eq/CK Eq, Aux Eq/ABA, CK Eq/ABA; Table 2) show that lower Aux level in comparison with CK and ABA levels as well as lower value of CK Eq/ABA favoured embryo-like structure formation and green plant regeneration ability. Another key factor was a proper balance between endogenous Auxs in anthers and exogenous Auxs supplied by culture media. To the best of our knowledge, this is the first such detailed examination of hormonal regulation of ME in triticale anther cultures. The results obtained so far widen our knowledge of the hormonal background underlying effective embryogenic development of microspores, though unambiguous explanation of the phytohormonal crosstalk needs further investigation.

## Electronic supplementary material

Below is the link to the electronic supplementary material.

Online Resource 1 The chromatogram of KIN-^15^N (heavy nitrogen labelled KIN—retention time RT=18 min) used as internal standard (a), external standard (KIN—Olchemim Ltd, Olomouc, Czech Republic) (b) and the substance identified as KIN in the samples (RT=18 min) (c). HPLC analyses were performed with the use of Agilent Technologies 1260 equipped with Agilent Technologies 6410 Triple Quad LC/MS with ESI (Electrospray Interface) (TIFF 170 kb)

Online Resource 2 The mass spectra of the external standard (KIN—Olchemim Ltd, Olomouc, Czech Republic) (a) and the substance identified as KIN in the samples (b). The most abundant secondary ions produced in Multiple Reaction Monitoring mode were used for additional qualification: ion 81.2 produced from ion 220.2 (marked as 220.2→81.2), ion 148.3 produced from ion 220.2 (220.2→148.3) and the ion 188.3 produced from ion 220.2 (220.2→188.3) (TIFF 156 kb)
