# Supplementary figures and images for: Hormonal requirements for effective induction of microspore embryogenesis in triticale (× Triticosecale Wittm.) anther cultures
Source: Plant Cell Rep. 2014 Sep 27;34(1):47–62. doi: 10.1007/s00299-014-1686-4 (PMC4282712; doi:10.1007/s00299-014-1686-4)

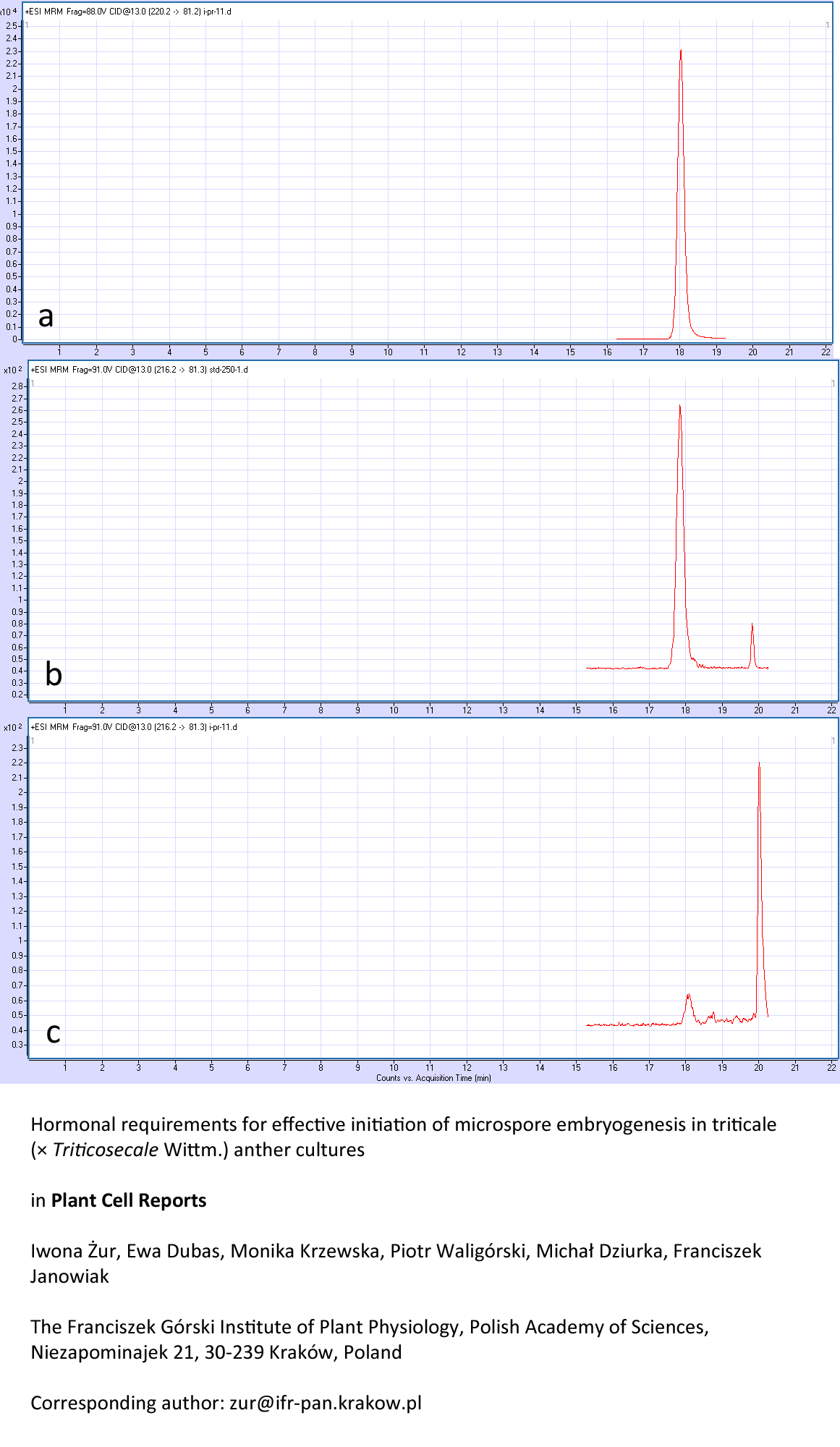

Supplement: Supplementary file 1 — Online Resource 1 The chromatogram of KIN-15N (heavy nitrogen labelled KIN—retention time RT=18 min) used as internal standard (a), external standard (KIN—Olchemim Ltd, Olomouc, Czech Republic) (b) and the substance identified as KIN in the samples (RT=18 min) (c). HPLC analyses were performed with the use of Agilent Technologies 1260 equipped with Agilent Technologies 6410 Triple Quad LC/MS with ESI (Electrospray Interface) (TIFF 170 kb) [file 299_2014_1686_MOESM1_ESM.tif]

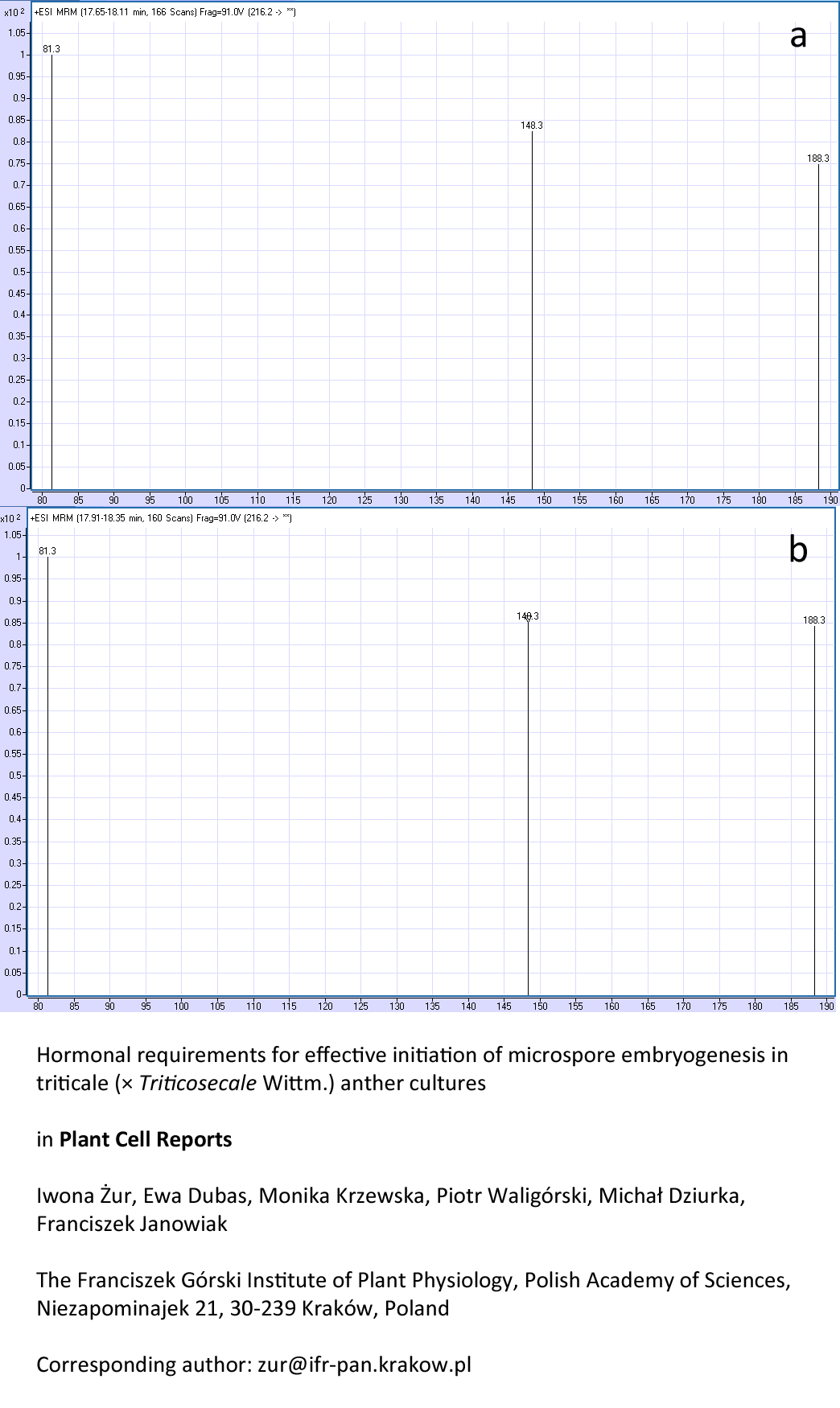

Supplement: Supplementary file 2 — Online Resource 2 The mass spectra of the external standard (KIN—Olchemim Ltd, Olomouc, Czech Republic) (a) and the substance identified as KIN in the samples (b). The most abundant secondary ions produced in Multiple Reaction Monitoring mode were used for additional qualification: ion 81.2 produced from ion 220.2 (marked as 220.2→81.2), ion 148.3 produced from ion 220.2 (220.2→148.3) and the ion 188.3 produced from ion 220.2 (220.2→188.3) (TIFF 156 kb) [file 299_2014_1686_MOESM2_ESM.tif]
